# Dr. Henry Baird Favill

**Published:** 1916-04

**Authors:** 


**Dr. Henry Baird Favill** of Chicago, Chairman of the Council of Public Health and Public Sanitation of the A. M. A., died Feb. 20 at Springfield, Mass., of pneumonia.

